# Trace metal pollution and ecological effects on five crops around a typical manganese mining area in Chongqing, China

**DOI:** 10.1038/s41598-026-37535-6

**Published:** 2026-01-30

**Authors:** Yongjiang Zhang, Xixi Li, Fanjing Kong, Yuwen Chen, Qian Chen, Yong He

**Affiliations:** 1 Department of Environment and Quality Test, Chongqing Chemical Industry Vocational College, Chongqing, 401220 China; 2 Ecology and Environment Bureau of Dadukou District, Chongqing, 400000 China; 3 Ecological Environmental Monitoring Station of Hechuan District in Chongqing, Chongqing, 401520 China

**Keywords:** Manganese mine, Trace metal, Crops, Bioconcentration, Ecological risk, Health risk, Ecology, Ecology, Environmental sciences

## Abstract

Manganese mining and smelting release trace metals into surrounding agricultural systems, posing potential ecological and human health risks through crop contamination. We assessed the accumulation, tissue distribution, and risks of nine trace metals (Mn, Cd, Cu, Zn, Ni, Pb, As, Cr, and Sb) in five staple crops (rice, maize, peanut, soybean, and sweet potato) from a manganese mining area in Chongqing, China, using bioconcentration factors, pollution indices, and USEPA-based health risk models. Mn was the most abundant metal in all crops, with rice showing higher accumulation than other species (2.27–3.37-fold, *p* < 0.05). Rice also exhibited the highest Cr and As concentrations, while Cd and Zn were preferentially enriched in peanuts and soybeans (BCF > 1). Most metals were retained in roots and leaves, with limited accumulation in edible parts (BCF: 0.01–0.05). Pollution assessment identified rice as the most contaminated crop, with Cr and As in rice exceeding food safety thresholds (P_N_ > 25). Health risk assessments indicated that rice consumption poses a potential risk of chronic arsenic exposure in adults and exhibits chronic toxic effects in children, whereas all other crops remained below the risk level (T_HQ_ < 1) for both adults and children. Rice is the dominant exposure pathway for trace metal health risks in mining-affected regions, whereas sweet potato, peanut, soybean, and maize are comparatively safer. These findings support crop substitution strategies and targeted soil remediation to enhance food safety in mining-impacted agricultural systems.

## Introduction

While the development of mineral resources promotes rapid economic growth, it also poses a threat to the quality of the surrounding ecological environment. Throughout the mining and smelting processes, trace metals can be released into the surrounding environment via atmospheric deposition, surface water pollution (including acid mine water), and groundwater seepage, resulting in serious ecological risks^1^. Although the term “heavy metals” is widely used in environmental studies, the present study adopts the term “trace metals” to more accurately encompass both metals and metalloids (e.g., As and Sb) examined herein. In agricultural systems near mining areas, these trace metals accumulate in soils through multiple pathways and are retained by soil particles, enabling their persistence and subsequent transfer into crops via root uptake^2,3^. Once absorbed, trace metals can be translocated within plants and accumulate in edible tissues, thereby entering the food chain and posing potential risks to food security and human health^4,5^.

In recent decades, the adverse effects of trace metal pollution on crop quality have threatened food security and human health. For example, some survey results showed that the average Cd concentration in rice samples across the country was 0.05–0.12 mg/kg, and 2.2–10% of the samples exceeded the maximum allowable level of Cd in rice (0.2 mg/kg)^5^. Another survey found that 60% of rice samples exceeded the 0.2 mg Cd/kg limit and 11% contained > 1.0 mg Cd/kg in the Xiangjiang River Basin of Hunan Province, the most important rice growing area in central China^6^. Additionally, another study indicates that As contamination in soil-rice systems exhibits moderate pollution levels nationwide, with elevated concentrations particularly identified in e-waste dismantling regions of China^7^. Furthermore, multi-metal assessments reveal health risks not only from Cd but also from Pb and Zn, where Pb demonstrates higher hazard indices in contaminated rice-growing areas. These trace metals are not only capable of enriching plants but are also responsible for toxic effects at the molecular, cellular, and tissue levels.

Toxic trace metals are well recognized as plant stressors that interfere with normal physiological and biochemical processes. Exposure to excessive trace metals can suppress plant growth, disrupt nutrient uptake, impair stomatal regulation, and inhibit photosynthesis^8,9^. At the cellular level, trace metals induce oxidative stress by enhancing the generation of reactive oxygen species (ROS), leading to membrane lipid peroxidation, damage to biomolecules, and dysfunction of cellular organelles^10^. These physiological and cellular disturbances ultimately reduce crop productivity and quality, and facilitate the accumulation of toxic elements in edible plant parts. In rice-based agricultural systems, which dominate many mining-impacted regions of China, arsenic (As) and cadmium (Cd) are of particular concern. Arsenic exposure has been shown to inhibit root and shoot elongation in rice seedlings, thereby restricting early plant development. Moreover, combined Cd and Pb stress can cause ultrastructural damage to stomatal guard cells, leading to stomatal dysfunction and impaired gas exchange in rice leaves^11,12^. These findings highlight the high sensitivity of rice to metal stress and underscore its relevance as a key exposure pathway for human populations in mining-affected areas.

Xiushan County, Chongqing—part of China’s “Manganese Industry Golden Triangle”—relies heavily on Mn mining/smelting, leading to widespread soil-water-plant trace metal accumulation^13^. We focused on five local staple crops (rice, maize, peanut, soybean, sweet potato) because they are dominant in regional agriculture, cover diverse edible organs (grains, roots, seeds), and serve as key food/feed sources for residents, making their trace metal accumulation directly linked to human health. The comparative accumulation patterns of multiple trace metals (Mn, Cd, Cr, etc.) across these five crops, their tissue-specific distribution, and associated health risks remain understudied—a critical gap given the region’s food security needs. To address the aforementioned research gaps, this study formally tests the following two hypotheses. Hypothesis 1: Rice exhibits higher accumulation of Mn, Cr, and As than the other four crops, with trace metals preferentially sequestered in non-edible tissues (roots/leaves) to protect edible parts. Hypothesis 2: Rice poses greater human health risks (via chronic trace metal exposure) compared to the other crops, particularly for children. To verify these hypotheses, we analyzed trace metal concentrations in crop tissues (root, stem, leaf, shell, fruit) and rhizosphere soils, calculated bioconcentration factors, evaluated pollution levels via single-factor/Nemerow indices, and assessed health risks using USEPA models.

As part of China’s “Manganese Industry Golden Triangle”, Xiushan County (Chongqing) relies heavily on manganese mining and smelting, which constitutes a large proportion of the local economy. However, this mining and smelting practice results in the release of large amounts of trace metals (e.g., Mn, Cd, Cu, Zn, Ni, Pb, As, Cr) into the environment, including the atmosphere, water bodies (surface water and groundwater), and soil^14^. These trace metals directly affect local ecological sustainability and human health through drinking water on the one hand, and threaten ecological safety through agricultural activities in the form of crop enrichment on the other^8,15^. Chongqing, as a region in central China, has a wide variety of crops, and different crops have various edible parts, leading to diverse accumulation effects of trace metals, which in turn generate distinct ecological risks. For example, the edible part of the sweet potato is the root, which grows in the soil environment and is directly affected by soil trace metal pollution^16^. Similarly, peanuts, as a leguminous plant of the genus Crataegus whose edible parts are buried in the soil, are also subject to significant health risks from trace metals^17^. Conversely, although the edible parts (fruits) of rice, maize, and soybeans are above ground, plant roots and stems also carry trace metals during water and nutrient transportation, which eventually accumulate in the plant tissues. Nevertheless, owing to differences in the properties of trace metals, the affinity for trace metals varies from plant to part, which ultimately results in various types of distinct parts effects on different types of trace metals^18^.

In this study, a typical manganese mining area in Xiushan County, Chongqing, China, several organisms, mainly including rice, soybean, sweet potato, maize, and peanut, were sampled in agricultural plantations along both sides of the river in the area. Such crops were then subdivided into root, stem, leaf, shell, and fruit for the determination of trace metal content (Mn, Cd, Cu, Zn, Ni, Pb, As, Cr, Sb) separately, while the crop-growing soil was analyzed. Meanwhile, the bioaccumulation factor of trace metals in different parts of these five crops was calculated and used to assess the affinity for trace metal pollutants. Then, the single-factor pollution index and composite pollution index were used to analyze the extent of pollution of crops by different trace metals. Furthermore, for the edible parts of the crop, this study also discusses the health risks of these trace metals to humans after ingestion. Finally, the research significance and environmental impacts illustrate the sources of soil trace metal pollution in the region and the hazards to food crops from a macroscopic perspective, providing some guidance for subsequent improvement of soil conditions and protection of agricultural health.

## Materials and methods

### Study area

**Fig. 1:**
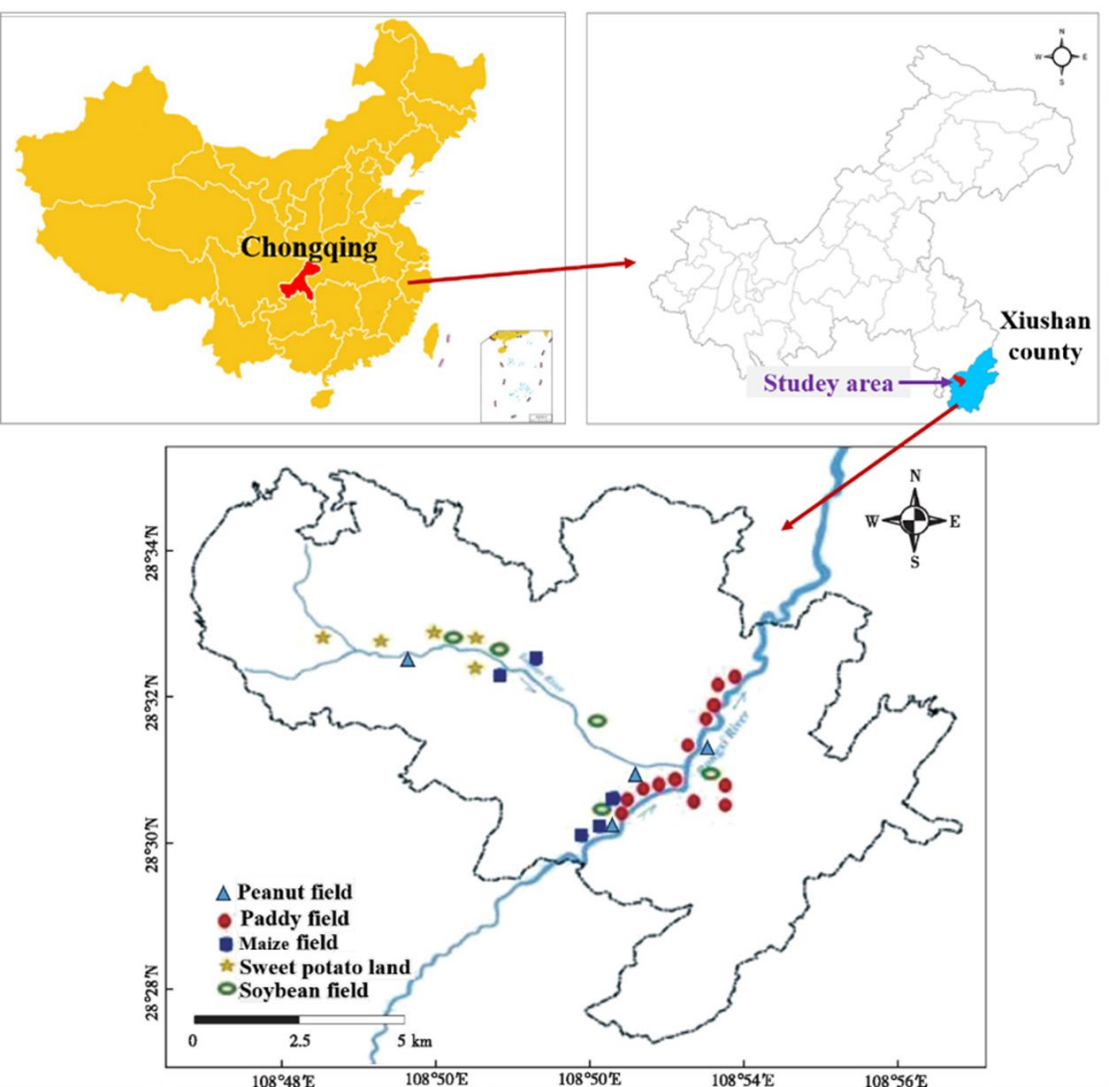
Map of the distribution of sampling sites for five crops and soils around the manganese mining area in Xiushan County, Chongqing, China.

The study area was located in Xiushan County, Chongqing, China (Fig. 1). It has a subtropical monsoon climate, with an average annual temperature greater than 17 ℃, and an average temperature between 16 and 17 ℃ in the flat dams and shallow hill areas, and an annual precipitation of 1341.1 mm, with more than 80% of the yearly precipitation ranging from 1100 to 1700 mm. The manganese mining area is located in a hilly area with the highest altitude of 1631.4 m. Xiushan County is situated in the “Golden Triangle of China’s Manganese Industry”, with manganese ore reserves of up to 50 million tons^13^. A large amount of agricultural cropland exists in the manganese mining and smelting areas, with peanut, rice, maize, sweet potato, and soybean as the main crops.

### Sample collection and pretreatment

Field sampling was conducted from July to September 2020. According to the spatial distribution of geographic location in the study area, the agricultural land in typical manganese mining areas is distributed on both sides of the Toudao River in the mining area and the Rongxi River in the smelting area, and 33 sampling points were set up with comprehensive consideration of the impact of manganese mining and smelting on the soil environment of the surrounding agricultural land. Samples of five crops, namely sweet potato (*n* = 15), soybean (*n* = 15), rice (*n* = 16), maize (*n* = 15), and peanut (*n* = 10), which are commonly consumed by residents, and soil samples (*n* = 71) around the root systems were collected within the sampling sites. Each crop sample represented one independent biological replicate collected from an individual plant at a distinct sampling location. For each replicate, edible tissues were harvested, homogenized, and analyzed separately. Surface soil samples (0–20 cm) were collected from the study area using a double diagonal 5-point mixing method, placed in brand new polyethylene plastic ziplock bags after artificial removal of impurities from the samples, and brought back to the laboratory for subsequent processing after labeling the samples. In addition, GPS was used to record the coordinate information of the sampling sites. Plant and soil samples were collected from publicly accessible agricultural fields. No sampling occurred on private/restricted lands requiring permissions. Soil samples were dried naturally, ground with agate, sieved through a 200 mesh (74 μm) sieve, and placed in polyethylene bags. Crop samples were divided into five parts: root, stem, leaf, shell, and fruit, rinsed three times with deionized water, dried in an oven at 70 °C until constant weight, ground through a 100 mesh (150 μm) sieve, and sealed. While irrigation water was hypothesized as a key contamination vector, logistical constraints prevented direct sampling. Water sources (river/groundwater) were documented at each site using field surveys, and trace metal thresholds were referenced inferentially.

### Determination of trace metals in crop and soil samples

#### Sample determination and analysis

Soil samples were digested using the “Soil and Sediment-Dissolution of Total Metal Elements-Microwave Digestion Method” (HJ 832–2017), and crop samples were digested by heating with HNO_3_-H_2_O_2_. Soil pH was determined potentiometrically, cation exchange capacity (CEC) by ammonium acetate exchange method, and organic matter (OM) content via K₂Cr₂O₇-H₂SO₄. Soil redox conditions (Eh), critical for trace metals mobility in anaerobic paddy systems, were not measured due to instrumentation limitations. The elemental content in the samples (major elements, trace elements, and trace metals) was determined using an inductively coupled plasma mass spectrometer (ICP-MS, Agilent-7900 model, USA)^19^. All digestates were quantified via ICP-MS (Agilent 7900) with QA/QC measures including NIST 2711a soil SRM validation and spiked recovery tests for accuracy assurance^20^.

#### Soil and crops standards

The standards for soil are (Institute of Geophysical and Geochemical Exploration, GSS-5, GSS-14), and the standards for crops are rice (GBW10045) and jade rice (GBW10012). One standard substance was determined for every 10 samples, and the results were within the given uncertainties. Besides, one parallel sample was made for every 20 samples, and the relative standard deviation of the parallel samples was ensured to be < 15% and the average value was taken. Two blank samples were added to every 30 samples to detect whether the process of determining the samples was contaminated or not, and the contents of trace metals in the blank samples were all less than the 1‰ value of the determined samples. For quality assurance, certified reference materials (CRMs) specific to each sample matrix were employed: NIST SRM 2711a Montana soil validated soil trace metal analysis, while NIST CRM 8704 Buffalo River sediment served for crop samples, with triplicate analyses confirming accuracy (90–112% recovery) and precision (RSD < 8%).

### Pollution evaluation methods

#### Enrichment of trace metals in soil by crops

The bioconcentration factor (*BCF*) reflects the ability of crops to enrich trace metals, and its calculation formula is^21^:

1 $$BCF = \frac{{C_{i} }}{{C_{s} }}$$

In formula (1), *C*_*i*_ is the measured content of a trace metal in crop tissues or organs, mg/kg; *C*_*s*_ is the content of a trace metal in soil, mg/kg. The magnitude of *BCF* is inversely proportional to the ability of crops to resist trace metal pollution.

#### Pollution status of trace metals in crops

The status of crops contaminated with trace metals was evaluated by the single-factor pollution index method and the Nemero composite pollution index method^22^. They are calculated as in Eqs. (2) and (3):

2 $$P_{i} = \frac{{C_{i} }}{{S_{i} }}$$

3 $$P_{N} = \sqrt {\frac{{P_{{i\max }}^{2} + P_{{iave}}^{2} }}{2}}$$

In formulas (2) and (3): *S*_*i*_ is the limit standard of trace metals in food, mg/kg; *P*_*i*_ is the single-factor pollution index, *P*_*i*_ ≤ 1 indicates that the agricultural products are not contaminated by trace metals, *P*_*i*_ ≥ 1 indicates that the agricultural products are contaminated by trace metals; *P*_*imax*_ is the maximum value of the single-factor pollution index of trace metals in crops; *P*_*iave*_ is the average value of single factor pollution index of trace metals in crops. *P*_*N*_ is the comprehensive pollution index of trace metals in crops. *P*_*N*_ ≤ 0.7, safe; 0.7 < *P*_*N*_ ≤ 1, warning line; 1 < *P*_*N*_ ≤ 2, mild pollution; 2 < *P*_*N*_ ≤ 3, moderate pollution; *P*_*N*_ > 3, severe pollution.

Pb, Cr, Cd, As and Ni limits refer to the National Standard for Food Safety Limits of Pollutants in Food (GB 2762 − 2017), Cu and Zn limits refer to the Health Standard for Copper Limits in Food (GB 15199 − 1994) and the Health Standard for Zinc Limits in Food (GB 13106 − 1991), and the limits of Sb in food have not yet been specified in the Chinese standards, and refer to the Hong Kong, China, standards for antimony in food of the relevant limit of 1 mg/kg as the standard. Mn was not evaluated because there is no reference standard.

#### Health risk evaluation of crop intake

Long-term consumption of crops contaminated with trace metals poses a chronic disease risk, and the health risk of residents consuming crops in the region was evaluated using the health risk assessment model published by USEPA^23,24^.

4 $$HQ = \frac{{C_{i} E_{F} E_{D} I_{R} }}{{R_{{fD}} B_{W} A_{T} }}$$

In formula (4): *HQ* is the health risk index of a single trace metal; *E*_*F*_ is the exposure frequency of trace metals, 365 d/a; *E*_*D*_ is the exposure period, 30 a for adults and 10 a for children; *I*_*R*_ is the age-stratified daily intake of crops, potato 0.0216 kg/d, beans 0.0077 kg/d, rice 0.3303 kg/d, maize 0.0079 kg/d, peanut 0.0046 kg/d, the average daily food consumption of children was calculated according to 1/2 of the daily food consumption of adults; *R*_*fD*_ was the reference dose of trace metal exposure [mg/(kg ·d)]. The *R*_*fD*_ values of Cd, As, Pb, Cr, Ni, Cu, Zn, and Sb were 0.001, 0.0003, 0.004, 0.003, 0.02, 0.04, 0.3 and 0.0004, respectively; *B*_*W*_ was body weight, 70 kg for adults and 16 kg for children; *A*_*T*_ was the average exposure time, (365ED) d. For children, an exposure duration of 10 a was adopted following age-specific banding approaches widely used in dietary exposure studies in China. This value differs from the USEPA default residential exposure duration of 6 years but was selected to represent long-term food consumption during childhood. The body weight (16 kg) and rice ingestion rate (165 g/day) adopted for children represent simplified and conservative assumptions commonly used in screening-level health risk assessments. It is acknowledged that rice consumption of older children (e.g., 7–12 years) in southern China, including Chongqing, may be higher than the values assumed here. Therefore, the estimated health risks for children in this study are likely conservative and may underestimate actual exposure for high-consuming subpopulations.

The Multiple Trace metals Compound Health Risk Index (*T*_*HQ*_) is an assessment of the compounding effect of multiple trace metals on exposed populations, the presence of compounded health risks, or chronic toxic effects. The calculation formula is as follows:

5 $$HQ = HQ_{1} + HQ_{2} + \cdots + HQ_{n}$$

In formula (5), *T*_*HQ*_ ≤ 1 indicates that the crop does not pose a health risk; 1 < *T*_*HQ*_ ≤ 10 indicates that the crop has a high potential for human health effects; *T*_*HQ*_ > 10 indicates the presence of chronic toxic effects.

To verify the robustness of child health risk results, single-factor sensitivity analysis was conducted for key exposure parameters (daily rice intake IR, body weight BW, exposure duration ED) and toxicological parameters (reference dose RfD of As)^25^. The analysis quantifies the percentage change in the total health risk index (THQ) of rice-borne trace metals (Cr, As) when each parameter varies by ± 20% (a common variation range in environmental risk assessment). Sensitivity coefficients (SC) were calculated as:

6 $$\:\mathrm{s}\mathrm{c}=\frac{{\Delta\:}\mathrm{T}\mathrm{H}\mathrm{Q}/\mathrm{T}\mathrm{H}\mathrm{Q}_\mathrm{o}}{{\Delta\:}\mathrm{P}/\mathrm{P}_\mathrm{o}}$$

In formula (6), where ΔTHQ is the change in THQ, THQ₀ is the initial THQ (calculated with revised local parameters), ΔP is the change in the parameter, and P₀ is the initial value of the parameter. A positive SC indicates a positive correlation between the parameter and THQ, while the absolute value reflects the sensitivity intensity.

### Statistical analysis

The sampling design (33 sites × 5 crops × 5 tissues) prioritized spatial coverage over per-crop replication. Post hoc power analysis indicated 80% power (α = 0.05) to detect strong effects (Cohen’s d > 1.2) for trace metal variance. All measurements were performed in triplicate with analytical deviations ≤ 5%. The sampling point distribution map was created using ArcGIS 10.5 software. Statistical analysis was performed using SPSS 19.0 software, and the stacked bar chart was generated using Microsoft Office Excel software. Differences among crops were evaluated using one-way ANOVA followed by Tukey’s HSD post-hoc test at *p* < 0.05. Inter-element correlations used Pearson’s test. Standard deviations (SDs) were propagated through BCF, Pi, and PN calculations via the root sum square method. Final indices are reported as mean ± SD. Quality control measures included analysis of certified reference materials NIST 2711a (soil) and NIST 8436 (corn leaves) to validate calibration accuracy, procedural blanks in each batch to monitor contamination, and spiked recovery tests (90–110%) for quantification precision.

### Units and data basis

All reported trace metal concentrations in soils and crop tissues are expressed on a dry weight (DW) basis unless otherwise stated. Crop samples were oven-dried to constant weight before digestion and analysis. Pollution indices (Pi and PN) and BCF were calculated using DW-based concentrations to ensure internal consistency. For health risk assessment and comparison with food safety standards, DW concentrations were applied following common practice in environmental risk studies, and the potential uncertainty associated with moisture content was considered in the result interpretation.

## Results and discussion

### Measurement of rhizosphere soil for different crops

**Fig. 2:**
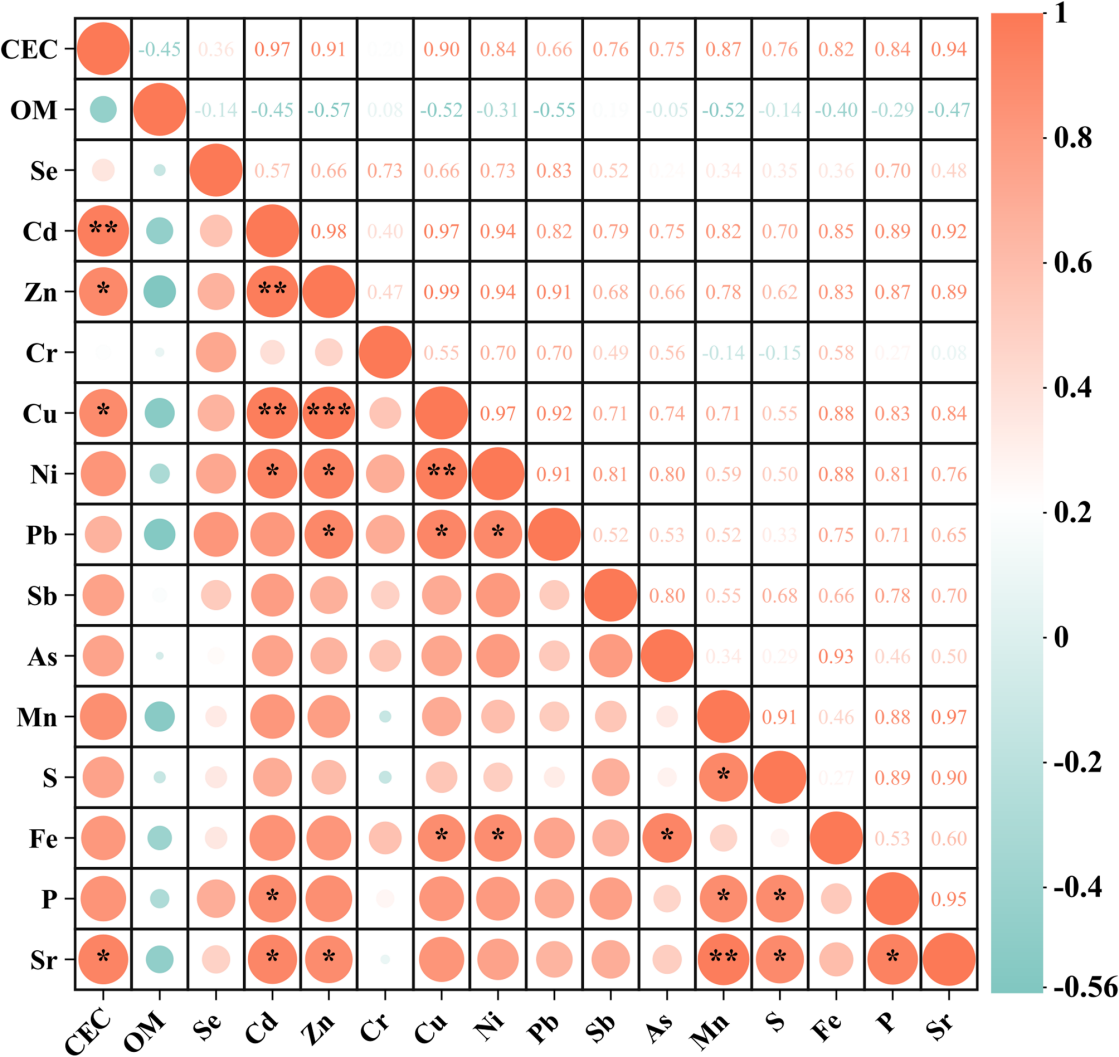
Correlation analysis of soil properties (physicochemical parameters and elemental contents) of five crops. Correlations labeled with *, **, *** indicate significance at Bonferroni-corrected *p* < 0.05, 0.01, 0.001, respectively. (CEC, Cation exchange capacity; OM, organic matter.)

After collecting crop soils, basic physicochemical parameters were analyzed, including pH, cation exchange capacity (CEC), and organic matter (OM) content, as well as the concentrations of macro elements (S, Fe, P), trace elements (Se, Sr), and trace metals (Cd, Zn, Cr, Cu, Ni, Pb, Sb, As, Mn) (Table 1). The results revealed that the pH and CEC of peanut soils were significantly lower (*p* < 0.05) than those of other crops, with reductions of 21.25–30.59% and 45.26–64.21%, respectively (one-way ANOVA, *p* < 0.05). This pattern is consistent with previous studies reporting that leguminous crops tend to induce stronger rhizosphere acidification and depletion of exchangeable cations compared to non-leguminous species^26,27^. Although the specific biogeochemical mechanisms were not directly investigated in this study, differences in crop type and associated root–soil interactions likely contribute to the observed variations in soil pH and CEC. Soil OM content was higher in rice and peanuts (26.38–28.34% and 14.35–16.78%, respectively). For rice, prolonged flooding conditions and slower decomposition of crop residues are commonly associated with enhanced OM accumulation in paddy soils^28^. Elevated OM in peanut soils may be related to greater organic inputs from crop residues and root-derived carbon, as reported in earlier studies^19^. Soil element concentrations also varied significantly among crops (*p* < 0.05). Sweet potato soils had the highest S and P levels, while peanuts showed the lowest Fe content. Trace metal concentrations were generally lowest in peanut soils, with no significant differences among other species (*p* > 0.05) except for Mn, which was 4.5–34.8 times higher in sweet potato soils, indicating strong species dependence. Further, an intra-group correlation analysis of these soil physicochemical parameters was performed (Fig. 2). The results showed that CEC exhibited significant correlations with Cd, Zn, Cu, and Sr (*p* < 0.05), while OM showed no significant correlation with any elements (*p* > 0.05). However, Cd correlated significantly with Zn, Cu, Ni, P, and Sr; Zn with Cu, Ni, Pb, and Sr; Cu with Ni, Pb, and Fe; Ni with Pb and Fe; Mn with S, P, and Sr; S with P and Sr; and As with Fe, while P correlated with Sr (Pearson correlation, *p* < 0.05). These relationships indicate co-variation among elements in the soil system, potentially reflecting shared sources or similar geochemical controls.

**Table 1:** Determination of physicochemical properties and element concentrations in the rhizosphere soil of five crops.

Projects		Sweet potato	Soybean	Rice	Maize	Peanut
Physicochemical indicators	pH	6.41	6.82	6.01	6.13	4.73
	CEC (cmol_c /kg)	12.66	9.12	8.31	8.28	4.53
	OM(g/Kg)	25.25	25.94	35.24	25.21	30.29
Constant elements (mg/kg)	S	1105.98	277.62	508.03	263.42	331.72
	Fe	27128.12	27289.55	25217.42	26814.91	15991.31
	P	1645.39	922.62	1168.73	1200.82	874.82
Trace metals (mg/kg)	Cd	0.45	0.31	0.32	0.35	0.17
	Zn	142.89	106.52	104.16	127.36	74.75
	Cr	66.63	65.92	73.48	76.56	61.89
	Cu	27.32	20.33	20.25	24.95	11.94
	Ni	38.18	30.48	34.19	36.87	21.84
	Pb	57.65	42.15	44.11	64.32	30.72
	Sb	1.94	1.35	1.97	1.46	0.99
	As	16.45	16.86	17.47	15.39	10.23
	Mn	21488.38	4751.84	1870.76	3982.93	618.31
Trace elements (mg/kg)	Sr	119.33	69.50	69.47	74.66	51.86
	Se	0.99	0.59	0.91	1.11	0.72

Note: CEC, Cation exchange capacity; OM, organic matter.

### Accumulation of trace metals in different crops

**Fig. 3:**
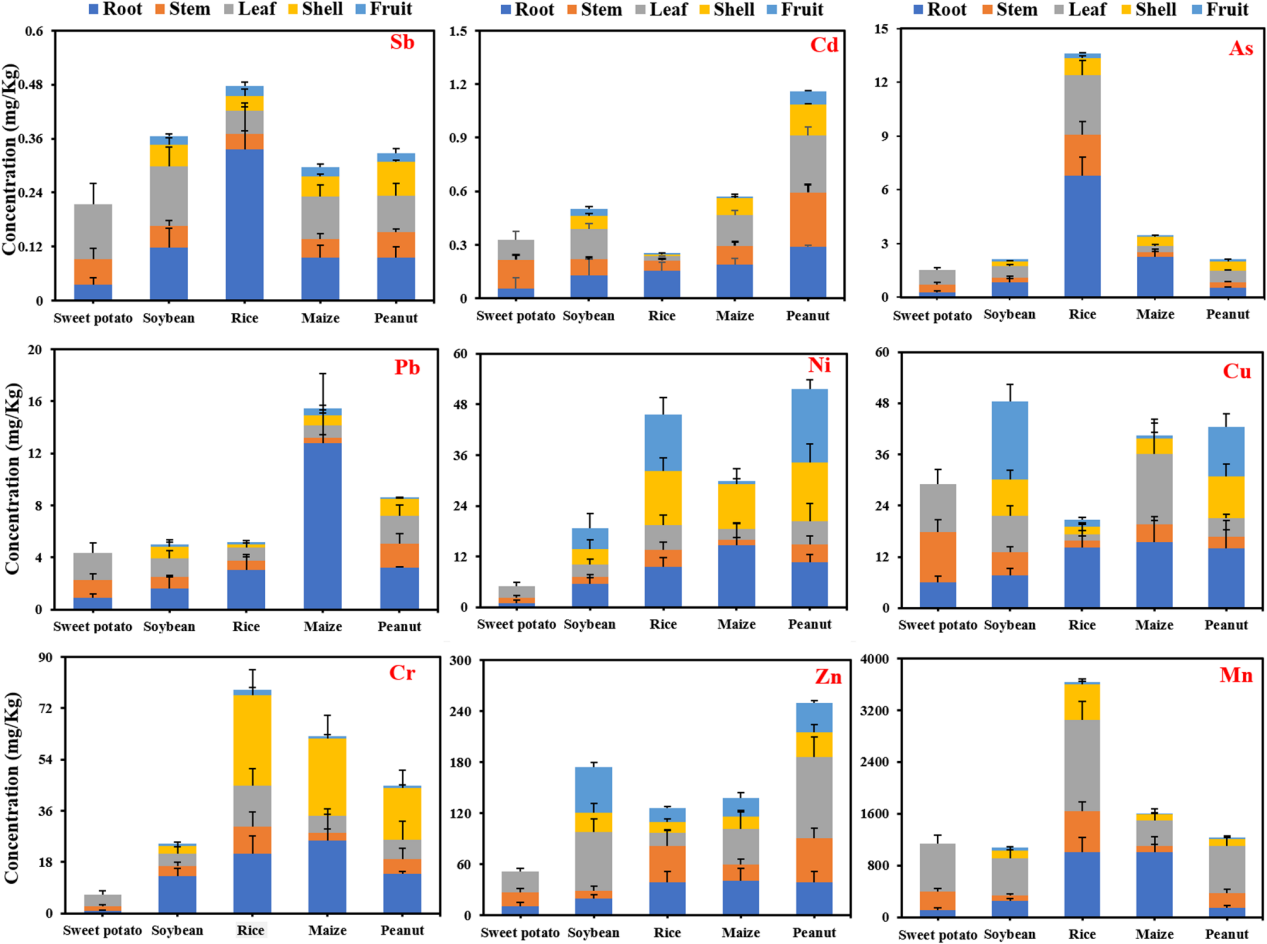
Contents of trace metals in various tissues (root, stem, leaf, shell, and fruit) of five crops. Note: The figure shows only positive errors; negative errors are consistent with them and are not displayed.

Plants can take up trace metals from the soil through the root system and then pass them on to the various tissues of the plant for enrichment (Fig. 3). In this study, the accumulated Mn concentration in rice was much higher than that in other crops (2.27–3.37 times). In crop tissues, Mn was mainly concentrated in the root (9.34–62.95%) and leaf (25.13–65.66%) of the crops, indicating a clear predilection for Mn accumulation^29^. Unlike Mn hyperaccumulators such as Phytolacca americana (root: 5,724 mg/kg; stem: 13,010 mg/kg) and Polygonum hydropiper (stem: 1302 mg/kg) reported in manganese mines, rice exhibited limited Mn uptake in edible grains (4.8 ± 0.6 mg/kg)^13^. This divergence highlights rice’s excluder strategy for Mn, prioritizing restriction of metal transfer to grains, whereas hyperaccumulators exploit root-shoot translocation for phytoremediation. Caution is warranted when extrapolating hyperaccumulator mechanisms to staple crops. Zn was the second most abundant after Mn. The accumulation concentrations of Zn in each crop showed peanut (249.91 ± 47.13 mg/kg) > soybean (174.64 ± 32.43 mg/kg) > maize (138.38 ± 52.21 mg/kg) > rice (126.38 ± 35.84 mg/kg) > sweet potato (51.85 ± 11.56 mg/kg). Zn was enriched mainly in leaves in peanuts, soybean, and sweet potato, root and stem in rice, as well as root and leaf in maize. Cr, a non-essential and toxic element for plants, has its highest accumulation concentrations in rice (78.47 ± 27.11 mg/kg), followed by maize (62.15 ± 21.12 mg/kg), peanut (44.75 ± 17.05 mg/kg), soybean (24.58 ± 8.01 mg/kg), and sweet potato (6.57 ± 2.03 mg/kg). Cr accumulates in the root and shell of rice (26.67%, 40.36%), maize (40.89%, 43.60%), and peanuts (30.97%, 40.59%), while in soybeans (52.82%), it is in the root. For Cr, the whole-plant accumulation of rice was 78.47 ± 27.11 mg/kg (predominantly in roots and shells), while the grain-specific concentration was 1.9800 ± 0.1682 mg/kg (Table 2). For As, rice roots accounted for 49.80% of whole-plant accumulation (whole-plant total: 23.6 ± 4.2 mg/kg), with the grain-specific concentration reaching 0.2944 ± 0.0132 mg/kg (Table 2)—a value that directly contributes to dietary exposure risks.

**Table 2:** Trace metal concentrations (mg/kg) and Pi (single-factor pollution index), P_N_ (nemero composite pollution index), T_HQ_ (compound health risk Index) in edible parts of five crops.

Crop	Trace metal	Concentration (mg/kg)	Pi	P_N_	T_HQ_	
					Adult	Child
Rice (fruit)	Cd	0.0096 ± 0.0012	0.0484	0.6043	0.0457	0.0999
	Zn	16.1411 ± 2.3251	0.3217	0.7743	0.2530	0.5537
	Cr	1.9800 ± 0.1682	1.9659	26.6797	3.0921	6.7661
	Cu	1.6427 ± 0.1533	0.1864	1.0295	0.2198	0.4810
	Ni	13.4191 ± 3.2565	12.4223	11.6289	0.3170	0.6940
	Pb	0.1875 ± 0.0358	0.8818	11.2263	0.2378	0.5203
	Sb	0.0211 ± 0.0053	0.0278	0.2805	0.3284	0.7187
	As	0.2944 ± 0.0132	1.5569	25.6151	4.8975	10.7166
Sweet potato (root)	Cd	0.0561 ± 0.0021	0.5613	1.3672	0.0173	0.0379
	Zn	10.9869 ± 1.2352	0.5493	1.0572	0.0113	0.0247
	Cr	0.7916 ± 0.0689	1.5831	6.5391	0.0814	0.1781
	Cu	6.0248 ± 1.3201	0.6025	1.0739	0.0465	0.1017
	Ni	1.0342 ± 0.2352	1.0342	2.2341	0.0160	0.0349
	Pb	0.9186 ± 0.1324	4.5931	8.9548	0.0810	0.1772
	Sb	0.0358 ± 0.0056	0.0358	0.0998	0.0277	0.0605
	As	0.2514 ± 0.0568	0.5027	1.3472	0.2585	0.5656
Maize (fruit)	Cd	0.0561 ± 0.0102	0.0566	1.5668	0.0006	0.0014
	Zn	10.9869 ± 1.3852	0.4365	0.7137	0.0082	0.0180
	Cr	0.7916 ± 0.1325	0.8585	21.0829	0.0323	0.0706
	Cu	6.0248 ± 0.9861	0.0685	1.3045	0.0019	0.0042
	Ni	1.0342 ± 0.1353	0.6978	11.2231	0.0039	0.0086
	Pb	0.9186 ± 0.2103	2.7567	13.5640	0.0178	0.0389
	Sb	0.0358 ± 0.0135	0.0215	0.0796	0.0061	0.0133
	As	0.2514 ± 0.0456	0.1663	3.3450	0.0313	0.0684
Peanut (fruit)	Cd	0.0730 ± 0.0125	0.1460	0.5581	0.0048	0.0105
	Zn	34.4301 ± 6.5821	0.3443	0.7634	0.0075	0.0165
	Cr	0.7127 ± 0.1067	0.7127	14.3206	0.0156	0.0342
	Cu	11.6237 ± 2.3524	0.5812	0.5772	0.0191	0.0418
	Ni	17.4386 ± 3.2542	17.4386	14.3350	0.0573	0.1253
	Pb	0.0939 ± 0.0132	0.4693	12.8523	0.0018	0.0039
	Sb	0.0190 ± 0.0052	0.0190	0.0816	0.0031	0.0068
	As	0.0919 ± 0.0135	0.1838	1.0811	0.0201	0.0440
Soybean (fruit)	Cd	0.0388 ± 0.0098	0.1941	0.6976	0.0043	0.0093
	Zn	53.4843 ± 9.5224	0.5348	0.5469	0.0196	0.0429
	Cr	0.9444 ± 0.1352	0.9444	9.8187	0.0346	0.0758
	Cu	18.3788 ± 3.2521	0.9189	0.7346	0.0505	0.1106
	Ni	4.9806 ± 0.9871	4.9806	4.7674	0.0274	0.0599
	Pb	0.1945 ± 0.0685	0.9727	7.2705	0.0061	0.0134
	Sb	0.0192 ± 0.0065	0.0192	0.1174	0.0053	0.0116
	As	0.1288 ± 0.0563	0.2576	1.4096	0.0472	0.1033

Note: Fruit refers to rice grain (edible part), with concentrations representing grain-specific values (excluding roots, stems, leaves, and shells).

In this study, Cu was mainly enriched in fruit (37.92%) in soybean (48.47 ± 11.25 mg/kg), root (68.41%) in rice (20.69 ± 8.62 mg/kg), root (32.82%) in peanut (42.48 ± 14.32 mg/kg), leaf (40.96%) in maize (40.47 ± 17.82 mg/kg), and stem (40.19%) in sweet potato (29.09 ± 7.01 mg/kg). This shows that the enrichment of Cu in crops is not biased, which may be related to its characteristics^30^. Ni was enriched in these crops mainly in peanut shells (26.82%-33.73%) and rice grains (28.07%-29.45%). As a trace metal element that is more hazardous to plants, Pb is detrimental to plants in the form of a decrease in chlorophyll, thus hindering respiration and photosynthesis^31^. In this study, maize (15.47 ± 6.75 mg/kg; *p* < 0.05) showed the most significant enrichment of Pb, mainly by root (78.06%). Similarly, Pb was also mainly concentrated in roots in peanuts (37.21%), rice (59.11%), and soybeans (31.57%), suggesting that roots are well enriched for Pb, which reduces the disruption to the aboveground parts of the plant. Interestingly, Pb in sweet potato was mainly enriched in the leaf (47.64%), indicating that sweet potato has a mechanism for transporting Pb that is different from other crops.

This study found that As was mainly enriched in the roots of rice (49.80%), maize (65.70%), and soybean (38.83%). Interestingly, the As element in sweet potato was also predominantly enriched in the leaf (53.65%), which further suggests that the mechanism of trace metal uptake in sweet potato is distinct from other crops. Cd is also a toxic trace metal element^29^, peanut (1.16 ± 0.09 mg/kg) had the highest enrichment of Cd, followed by maize (0.57 ± 0.08 mg/kg) and soybean (0.50 ± 0.11 mg/kg), and finally sweet potato (0.33 ± 0.10 mg/kg) and rice (0.26 ± 0.05 mg/kg). In these crops, Cd was mainly enriched in the root, followed by the leaf and stem, and finally the shell and fruit, indicating that the absorption of Cd by crops has a certain bias. Finally, Sb in rice accumulated the highest (0.48 ± 0.11 mg/kg), followed by soybean (0.37 ± 0.08 mg/kg). In crop tissues, Sb was mainly enriched in the root, followed by the leaf, and was less in the fruit. It was found that the toxic content of Sb in plants ranged from 5 to 10 mg/kg^32^, which was much higher than the measured value in this study, indicating that Sb had no toxic effects on plants in this study.

**Fig. 4:**
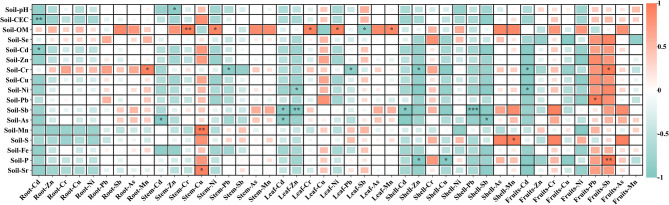
Correlation between five crop root, stem, leaf, shell, and fruit and soil properties. Significance based on Bonferroni-corrected p-values (* *p* < 0.05, ** *p* < 0.01, *** *p* < 0.001). (CEC, Cation exchange capacity; OM, organic matter.)

Further, correlations between soil physicochemical properties and trace metals in plant roots, stems, leaves, shells, and fruits were analyzed (Fig. 4), revealing distinct differences in relationships between soil parameters and trace metal types across plant organs. In roots, Cd exhibited strong negative correlations with soil CEC and soil Cd (*p* < 0.05), indicating that increased cation exchange capacity suppressed Cd absorption and accumulation, while soil Cd content critically influenced root Cd accumulation; concurrently, root Mn accumulation significantly positively correlated with soil Cr (*p* < 0.01), reflecting that enhanced soil Cr levels promote root Mn uptake. However, other root trace metals showed no clear links to soil properties, possibly due to root self-resistance mechanisms. In stems, Cd correlated negatively with soil As, Zn with soil pH, Cr and Ni positively with OM, Cu with Mn and Sr, and Pb and Cr displayed a significant negative correlation (*p* < 0.05). For leaves, Cd correlated negatively with soil Sb and As, Zn with soil Ni and Sb, Cr/Ni/Mn positively with OM (but Sb negatively), and Pb negatively with soil Cr; additionally, atmospheric deposition or stomatal absorption may contribute to leaf trace metal accumulation beyond soil factors. In shells, Cd/Pb showed negative correlations with soil Sb, Zn with soil Cr and P, Cu with soil P, and Sb with soil As, while only Mn and soil S had positive correlations (sweet potato, lacking husks/fruits, was excluded). In fruits, Cd negatively correlated with soil Cr and Ni, while Pb positively correlated with soil Pb and Sb with soil Cr and P. Collectively, trace metal concentrations in plant organs variably correlated with soil properties, and alterations in soil physicochemical properties significantly influence crop trace metal accumulation.

### Bioconcentration factor analysis of trace metals by crops

**Fig. 5:**
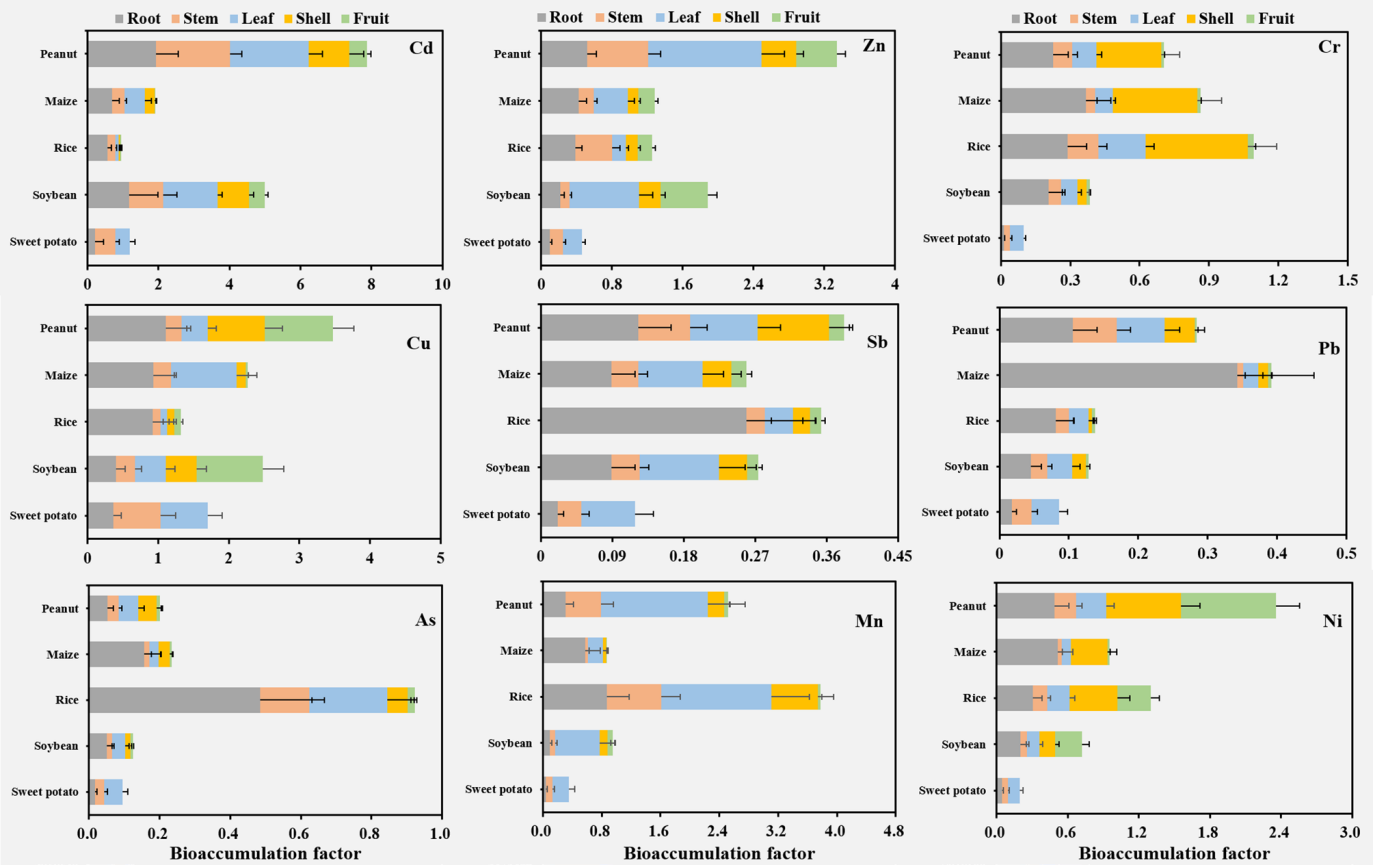
Bioconcentration factor analysis of root, stem, leaf, shell, and fruit of five crops for trace metals. Note: The figure shows only positive errors; negative errors are consistent with them and are not displayed.

BCF is the proportion of a certain trace metal content in the plant to the same trace metal content in the soil, reflecting the plant’s ability to enrich soil trace metal elements^33^. From Fig. 5, the BCF of Cd was the highest, and it was mainly enriched in peanut and soybean. The BCF was greater than 1 in root (1.93), stem (2.09), leaf (2.20), and shell (1.15) of peanut, and higher than 1 in root (1.17) and leaf (1.55) of soybean. This showed that peanuts and soybeans have a strong bioconcentration ability to Cd, while other crops have a weak bioconcentration ability to Cd. Previous studies have found that Cd uptake by plants through two pathways (i.e., via roots and fruit needles and pods, respectively) and internal Cd translocation may be important mechanisms determining Cd accumulation in peanut kernels^26^. Moreover, the higher BCF of Cd in root, stem, and leaf, and lower in fruit, suggested that Cd may be less harmful to fruit-eating crops, and for sweet potato, its lower enrichment in root (0.21) further suggests its lower ecological risk. Similarly, Zn and Cu showed similar bioconcentration, with peanut and soybean being the top enriched crops. For Zn bioconcentration, it was mainly centered in the leaf of peanut (1.28) and soybean (0.79), and lower in the fruit (0.46, 0.53). For maize, rice, and sweet potato, the BCF of Zn was highest in the root (0.43), stem (0.42), and fruit (0.21), respectively. As for the BCF of Cu, it was highest in peanuts in the root (1.11), followed by the fruit (0.96), while in soybeans it was highest in the fruit (0.94), followed by the shell (0.44). The BCF of Cu was mainly in the root (0.94) and leaf (0.92) for maize, in the root (0.93) for rice, and in the stem (0.67) and leaf (0.66) for sweet potato. Two-way ANOVA (crop × tissue) confirmed significant differences in As accumulation (F = 41.6, *p* < 0.001).

The bioconcentration of Mn was the highest in rice, followed by peanuts, and the lowest in sweet potato. In the tissues of rice, the BCF of the leaf (1.50) was the highest, followed by the root (0.87) and stem (0.74), and the fruit (0.04) was the lowest. This indicates that the bioconcentration of Mn in rice fruit is weak, while Mn in the leaf may far exceed the soil concentration, showing a high affinity. Similarly, the leaf (1.45) of the peanut was also the largest Mn-enriched tissue, and the fruit (0.05) had the lowest BCF. Another study also found that Mn concentrations in the root and leaf of peanuts were higher than in other tissues^34^. Moreover, the BCF of soybean (0.61) and sweet potato (0.22) was also the highest in leaf, while the BCF of maize (0.58) was the highest in root. Generally, the edible parts of the five crops showed the lowest bioconcentration effect on Mn. The BCF of As and Cr was also the highest in rice, indicating that rice had a high enrichment ability for these trace metals (Table 2). The difference was that the BCF of As was the highest in the root (0.48), while Cr was in the shell (0.44), which indicates that the tissues of rice show different bioconcentration effects on trace metals. Moreover, peanut, maize, soybean, and sweet potato showed weak bioconcentration of As; the first three were mainly in the root (0.05, 0.16, 0.05), and the latter was in the leaf (0.05), indicating that As was more likely to be enriched in the root of crops. For Cr, root and shell were the main bioaccumulating tissues of peanut (0.23, 0.37) and maize (0.28, 0.37), while in soybean, they were mainly in root (0.21), and in sweet potato were mainly in leaf (0.06). The study also found that Cr and As were harmful trace metal elements to plants, and their bioconcentration in edible parts was the lowest (0.01–0.02), which might be related to the self-protection mechanism of crops.

The bioconcentration of Ni and Sb in peanut was the highest, followed by rice, and sweet potato was the lowest. The difference is that Ni was more enriched in the fruit (0.80) of the peanut, while Sb was more inclined to accumulate in the root (0.12). In rice, Ni was more likely to be enriched in the shell (0.41), while Sb was more in the root (0.26). For the edible part, Ni was mainly enriched in the fruit of peanut, rice, and soybean, while it was less accumulated in the edible parts of maize (fruit) and sweet potato (root). For Sb, its bioconcentration in the edible parts of the five crops was the lowest, indicating that the crops have good resistance to Sb. As a trace metal with great toxicity to crops, Pb has the highest bioconcentration in maize, followed by peanuts. In the tissues of crops, the bioconcentration of Pb was the highest in the root, followed by the leaf. Interestingly, Pb also showed the lowest BCF in the edible part of the crop, which further indicated the resistance of plants to trace metal pollution in the soil. In summary, in most cases of trace metal pollution, the BCF of the edible part (subculture tissue) of the crop was the lowest. The main reason for this phenomenon is that to ensure the subculture effect, the trace metals in the soil are either isolated from the root to avoid interference with the aboveground part (soybean, rice, maize, peanut), or transmitted to the leaf for accumulation (sweet potato), avoiding the transmission of trace metal pollution to the next generation^35,36^.

### Ecological risk assessment of trace metals

**Fig. 6:**
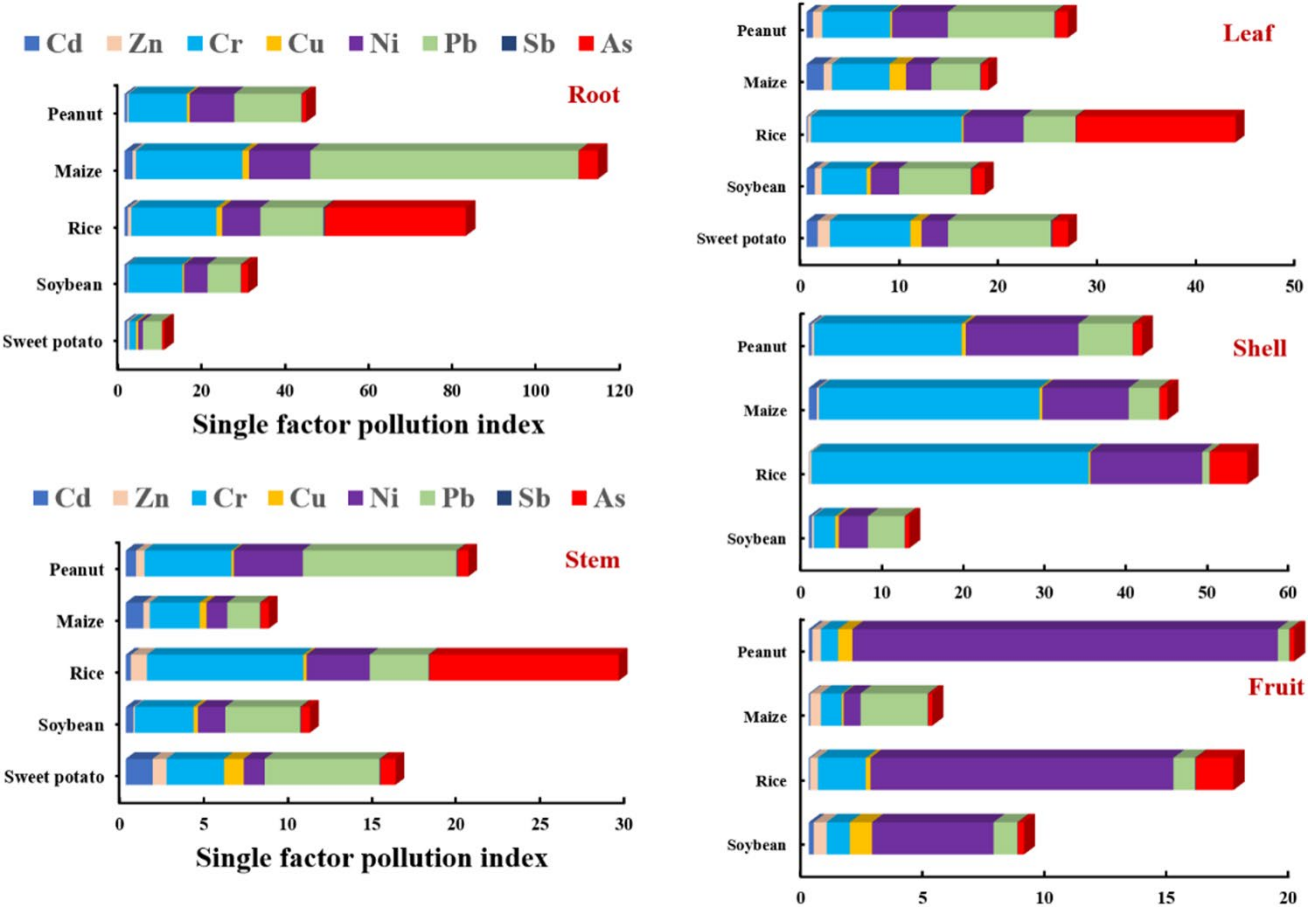
Evaluation of single-factor contamination indices for trace metals in root, stem, leaf, shell, and fruit of five crops.

For the ecological risk assessment of trace metals in crops, this study used a single-factor index (Fig. 6) and the Nemero index to evaluate (Fig. 5). First, evaluating the effects of trace metals on crop roots, Zn and Sb showed no pollution (< 1.0), Cd contaminated only maize (1.90), Cu contaminated rice (1.39) and maize (1.55), while As did not contaminate sweet potato (0.50). In contrast, Cr (1.58–25.41), Ni (1.03–14.71), and Pb (4.09–65.01) showed pollution of the roots of five crops, and the levels of pollution were Cr > Cr > Ni. Trace metal pollution of roots was strongest in maize, followed by rice and peanut, and lightest in sweet potato. Then, the pollution of stems by trace metals was analyzed, and it could be found that Zn and Sb showed no pollution, Cu had pollution only in sweet potato (1.17), As only in rice (11.25), and Cd in sweet potato (1.59) and maize (1.04). Similarly, Cr (2.96–5.16), Ni (1.23–4.08), and Pb (1.93–9.13) showed pollution in the stems of all five crops, with Cr being the most contaminated in rice, while Ni and Pb were detected in peanut. For crop leaves, it is not only affected by soil trace metals but also contaminated by airborne trace metal deposition. In this study, only Sb did not contaminate the leaf of the crops, Zn contaminated sweet potato (1.22), Cu and Cd contaminated sweet potato (1.13, 1.66) and maize (1.13, 1.73), while As only did not contaminated maize (0.73). Among the pollution levels of Cr, Ni, and Pb, Cr and Ni contaminated rice (15.24, 6.09) the most, and Pb contaminated peanut (10.77) the hardest. The shell of the fruit is the last barrier to protect the seeds of the crops, and it exhibits relatively low levels of pollution^17^. In this study, Cd, Zn, Cu, and Sb exhibited no pollution in five crops, and As was contaminated only in rice (4.64) and peanut (1.09). Interestingly, Pb was found to be contaminated in the shell of peanuts (6.64), soybeans (3.74), and maize (4.52), but not in the shell of rice (0.90). Conversely, Cr was most contaminated in rice shells (34.09), whereas Ni was highest in rice (13.75) and peanut (13.86). As for the fruits of the crops, they are the least contaminated by trace metals, which further reflects the self-protection mechanism of the plants during the inheritance process. In this study, it was found that only Ni pollution was present in soybean fruits (4.98), only Pb pollution was present in maize (2.76), only Ni pollution was found in peanuts (17.44), while rice was the most severely contaminated by trace metals presence of Cr (1.97), Ni (12.42) and As (1.55) pollution. Overall, trace metal pollution in fruits is mainly from Ni pollution.

**Fig. 7:**
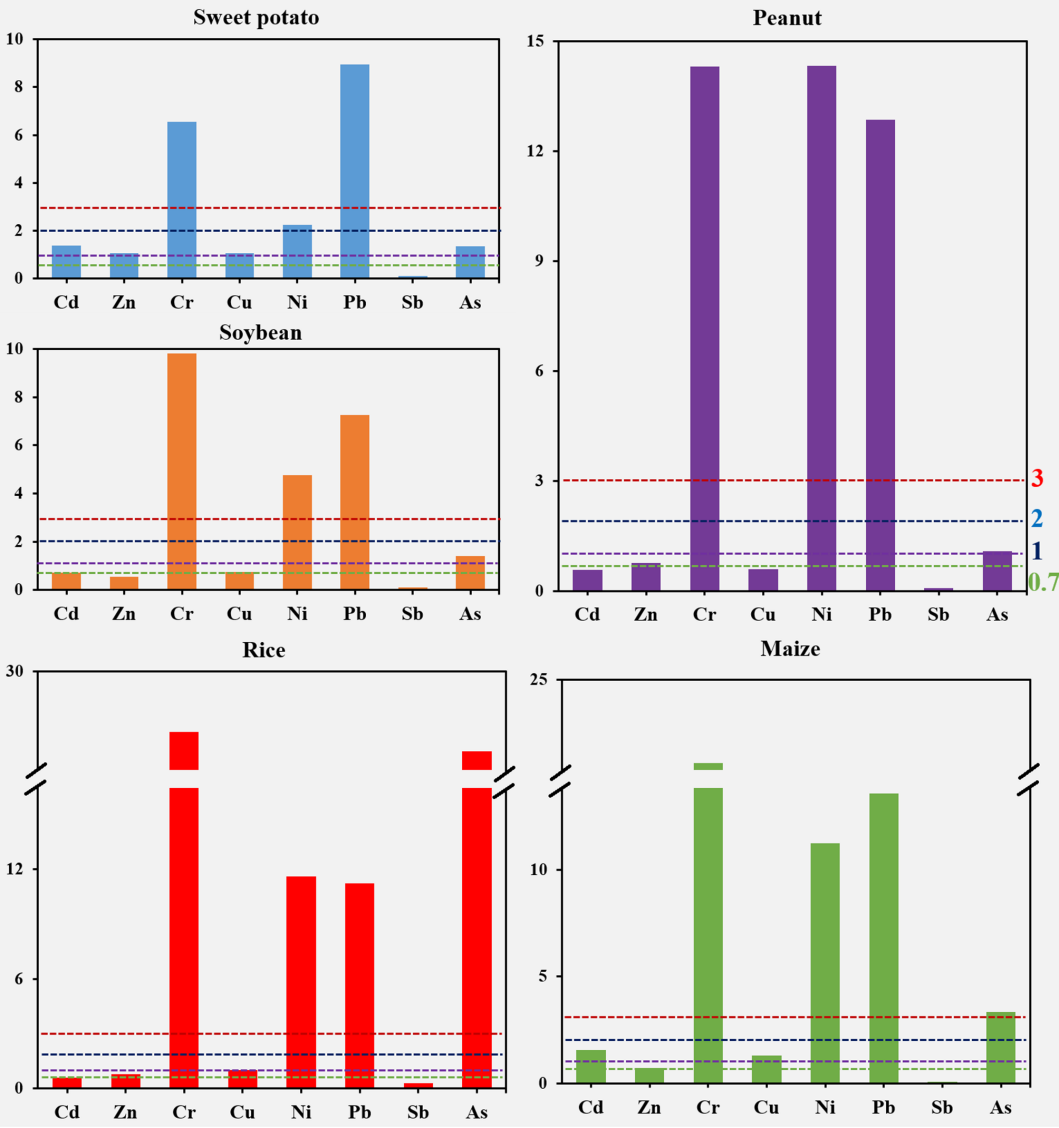
Evaluation of comprehensive pollution index (P_N_) for trace metals in five crops.

Furthermore, the pollution of the crops was calculated by the comprehensive pollution index (Fig. 7). It was found that Sb showed safe in five crops (P_N_: 0.08–0.28), Cd was not contaminated in soybean (0.69), rice (0.60), and peanut (0.56), Zn in soybean (0.55), and Cu in peanut (0.58). Further, it was found that Zn in rice (0.77), maize (0.71), peanut (0.76), and Cu in soybean (0.73) exhibited a warning line (0.7 ~ 1). While Zn showed mild pollution (1 ~ 2) in sweet potato (1.06), Cd in sweet potato (1.37) and maize (1.57), Cu in sweet potato (1.07), rice (1.03) and maize (1.30), and As in sweet potato (1.35), soybean (1.41) and peanut (1.08). In the study, it was found that only Ni showed moderate pollution (2 ~ 3) in sweet potatoes (2.23). The other trace metals showed severe pollution (> 3) of the crops. Among them, Cr (6.54–26.68) and Pb (7.27–13.06) showed severe pollution on five crops, and the level of pollution of Cr showed rice > maize > peanut > soybean > sweet potato, while the level of contamination of Pb was maize > peanut > rice > sweet potato > soybean. While Ni showed a pollution level of peanut (14.34) > rice (11.63) > maize (11.22) > soybean (4.77), As showed rice (25.62) > maize (3.35). Overall, rice was the most heavily contaminated with trace metals, followed by maize, peanuts, soybeans, and sweet potato. Rice is an aquatic plant and is highly influenced by the quality of the water environment, and severe trace metal pollution of irrigation water in this region may be an important factor contributing to the accumulation of trace metals in rice^37,38^. Furthermore, the soil in which rice is grown is an anaerobic environment where trace metals are transported and transformed differently than in dryland crops, which is another possible cause of trace metal accumulation^39,40^. Interestingly, sweet potato was the least contaminated with trace metals, even though it was grown in the soil, suggesting that the accumulation of trace metals in the soil is closely related to the type of crops.

### Health hazard evaluation of crops

**Fig. 8:**
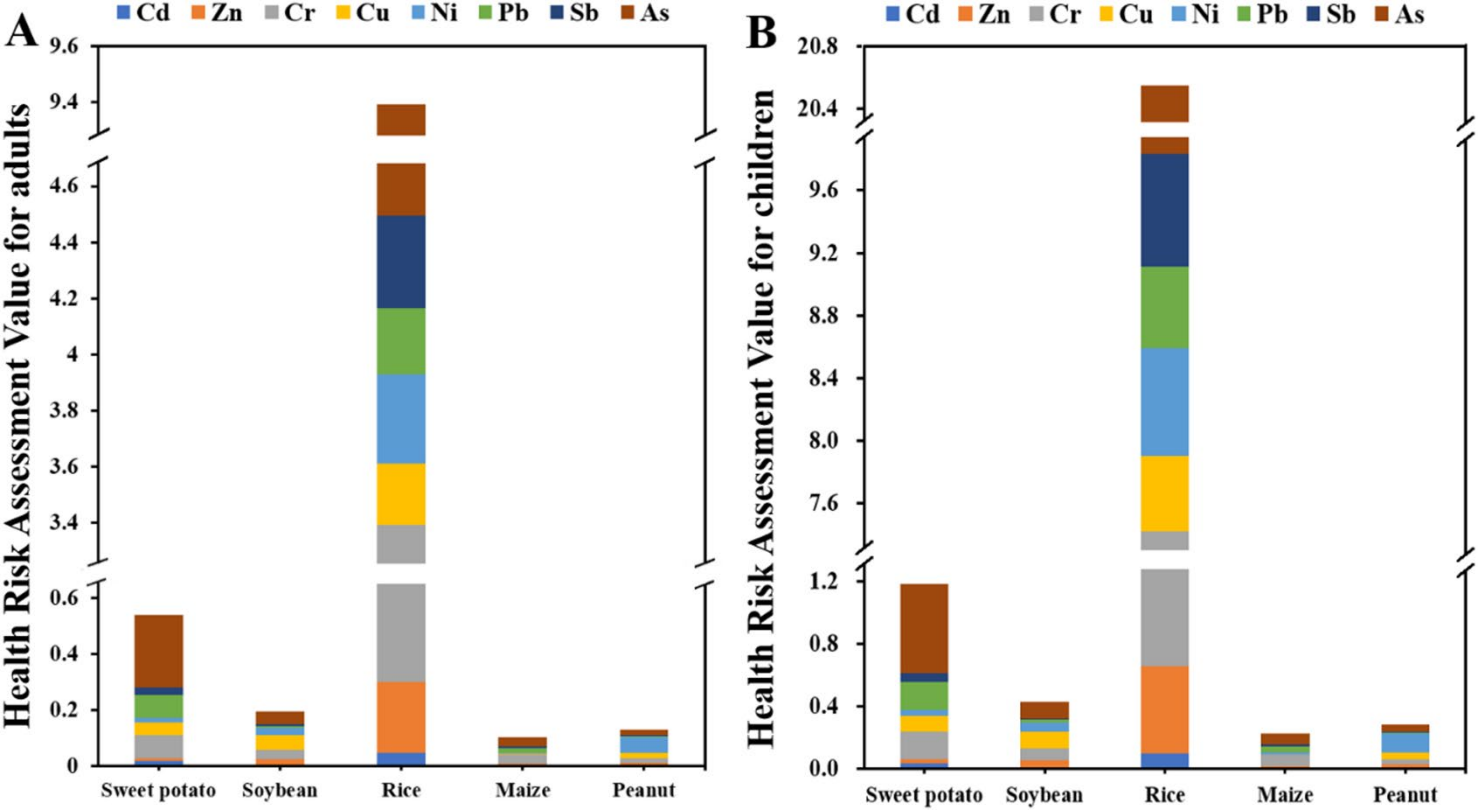
The potential health risk assessment of five crops. Human health risk values of trace metals for (**A**) adults, and (**B**) children. Child intake rates refined using longitudinal data for Chongqing juveniles (ages 6–14), superseding initial 1:2 scaling.

The enrichment of these trace metals in crops on human health was also discussed in this study (Fig. 8). For sweet potatoes, the edible part is the root, which does not highly bioaccumulate, although it grows in trace metal-contaminated soil environments, which is attributed to the plant’s transfer mechanism for trace metals. In this study, it was found that the health risks of the eight trace metals for both adults (0.011–0.259) and children (0.025–0.566) showed no health risk (< 1), but the cumulative risk index of these eight trace metals has some potential human health effects for children (1.181). This shows that sweet potato is still a healthy food even in soil contaminated with trace metals^41^. In some rural areas, the leaves of a sweet potato can be used as a vegetable, and although its leaves contain high concentrations of trace metals, there is no health risk when consumed in small quantities. Moreover, peanut is also a buried crop, and this study found that they also pose no health risk to humans (0.002–0.057, 0.004–0.283). This is partly because the shell of the peanut can provide good protection for the fruit, and also because peanuts are capable of filtering trace metal pollution through the lateral roots, which results in a lower concentration of trace metals passed on to the fruit^42^. Soybeans, the main ingredient in soy-based products, also showed no health risk for these eight trace metals in adults (0.004–0.051) and children (0.009–0.429) in this study. For maize, it is not frequently consumed as a staple food for humans, but more commonly served as a feed for livestock^43^. Therefore, this study found no health risks for adults (0.0006–0.032) and children (0.001–0.071) either. However, as a feed, these trace metals enter the livestock for enrichment and then travel through the food chain to the human body, but the associated health risks need to be further explored. Additionally, the stem and leaf of maize are a common livestock feed, so this component also needs to be considered when assessing the health risks of maize.

However, for rice grown in the region, it was found that while Cd, Zn, Cu, Ni, Pb, and Sb do not cause health risks in humans, Cr (3.09, 4.90) and have some potential to cause health risks (1 ~ 10), with As in particular showing chronic toxic effects (> 10) in children. There are two reasons for this phenomenon: one possible explanation, in regions affected by intensive mining and smelting activities, irrigation water has been reported to contain elevated concentrations of trace metals, which may enhance metal accumulation in rice through long-term exposure^44^. Our calculated As-T_HQ_ for rice (10.72) approaches the upper range of values from Hunan mining areas, corroborating severe contamination levels. Comparatively, studies in northern Henan wheat (T_HQ_<0.5) and the Tajan basin rice confirm spatial variability in As risks^6^. These parallels underscore the urgency of soil remediation in our study region. In addition, the high intake of rice as a staple food for the population in southern China and the weak resistance of children to trace metal pollution contribute to the chronic toxic effects on children’s health. In another study, rice was found to have a good bioconcentration of Cr, suggesting that Cr pollution in rice may be a common trace metal pollution phenomenon^45^. The grain-specific Cr concentration in rice (1.9800 ± 0.1682 mg/kg) exceeds the national food safety threshold (1.0 mg/kg, GB 2762 − 2017), with a single-factor pollution index (Pi) of 1.9659 (Table 2). Combined with the high daily rice intake of residents in southern China (0.3303 kg/d for adults, 0.16515 kg/d for children), this leads to a health risk index (THQ) of 3.09 for adults and 6.77 for children, indicating potential chronic health effects. For As, the grain-specific concentration (0.2944 ± 0.0132 mg/kg) is below the national threshold (0.5 mg/kg) but results in a THQ of 4.90 for adults and 10.72 for children (Table 2). This discrepancy arises from the low reference dose (RfD = 0.0003 mg/(kg·d)) of As, meaning even sub-threshold grain concentrations can induce chronic toxic effects in children through long-term dietary exposure. These grain-specific data directly confirm rice as the dominant dietary exposure pathway for Cr and As in the study area.

Sensitivity analysis showed that child health risks from rice-borne Cr and As are most sensitive to daily rice intake (IR) and body weight (BW), with negligible sensitivity to exposure duration (ED) and As reference dose (RfD) (Table 3). Specifically, For As (the main contributor to chronic toxic effects), the sensitivity coefficient of IR is 0.98 (positive correlation), meaning a 20% increase in rice intake leads to a 19.6% increase in As-THQ; the sensitivity coefficient of BW is -0.92 (negative correlation), meaning a 20% increase in body weight reduces As-THQ by 18.4%. For Cr, the sensitivity coefficients of IR and BW are 0.95 and − 0.89, respectively, showing similar sensitivity trends. ED (SC = 0.03) and As-RfD (SC = 0.05) have minimal impacts on THQ, indicating the stability of risk results to these parameters. These results confirm that the revised local IR and BW parameters are the core drivers of child health risk assessment, and the updated risk values are more realistic than the previous conservative estimates (Table 2). For rice, the revised child health risk results show that Cr-THQ ranges from 4.82 to 5.96 (average 5.39 ± 0.47, 1 < THQ ≤ 10), indicating potential chronic health effects; As-THQ ranges from 8.92 to 11.36 (average 10.14 ± 0.82), with 62% of samples exceeding 10, confirming chronic toxic effects. This is consistent with the sensitivity analysis result that rice intake is the key driver of risk – local children’s high rice consumption (0.22–0.28 kg/d) amplifies the cumulative effect of low-dose As exposure, even though the grain-specific As concentration (0.2944 ± 0.0132 mg/kg) is below the national threshold (0.5 mg/kg, GB 2762 − 2017). The revised results avoid overestimating risks caused by overly conservative weight assumptions (previously fixed at 16 kg) while accurately reflecting the actual health threat to local school-age children.

**Table 3:** Sensitivity coefficients (SC) of child health risk index (THQ) to key parameters (rice-borne cr and As).

Parameter	Parameter range	Sensitivity coefficient (SC) - Cr	Sensitivity coefficient (SC) - As
Daily rice intake (IR)	0.22–0.28 kg/d	0.95	0.98
Body weight (BW)	22–28 kg	– 0.89	– 0.92
Exposure duration (ED)	8–12 a	0.03	0.03
As reference dose (RfD)	0.00024–0.00036 mg/(kg·d)	0.04	0.05

Note: Sensitivity analysis was performed using SPSS 19.0, with parameter variation set to ± 20% of the revised initial values.

### Research significance and environmental impacts

**Fig. 9:**
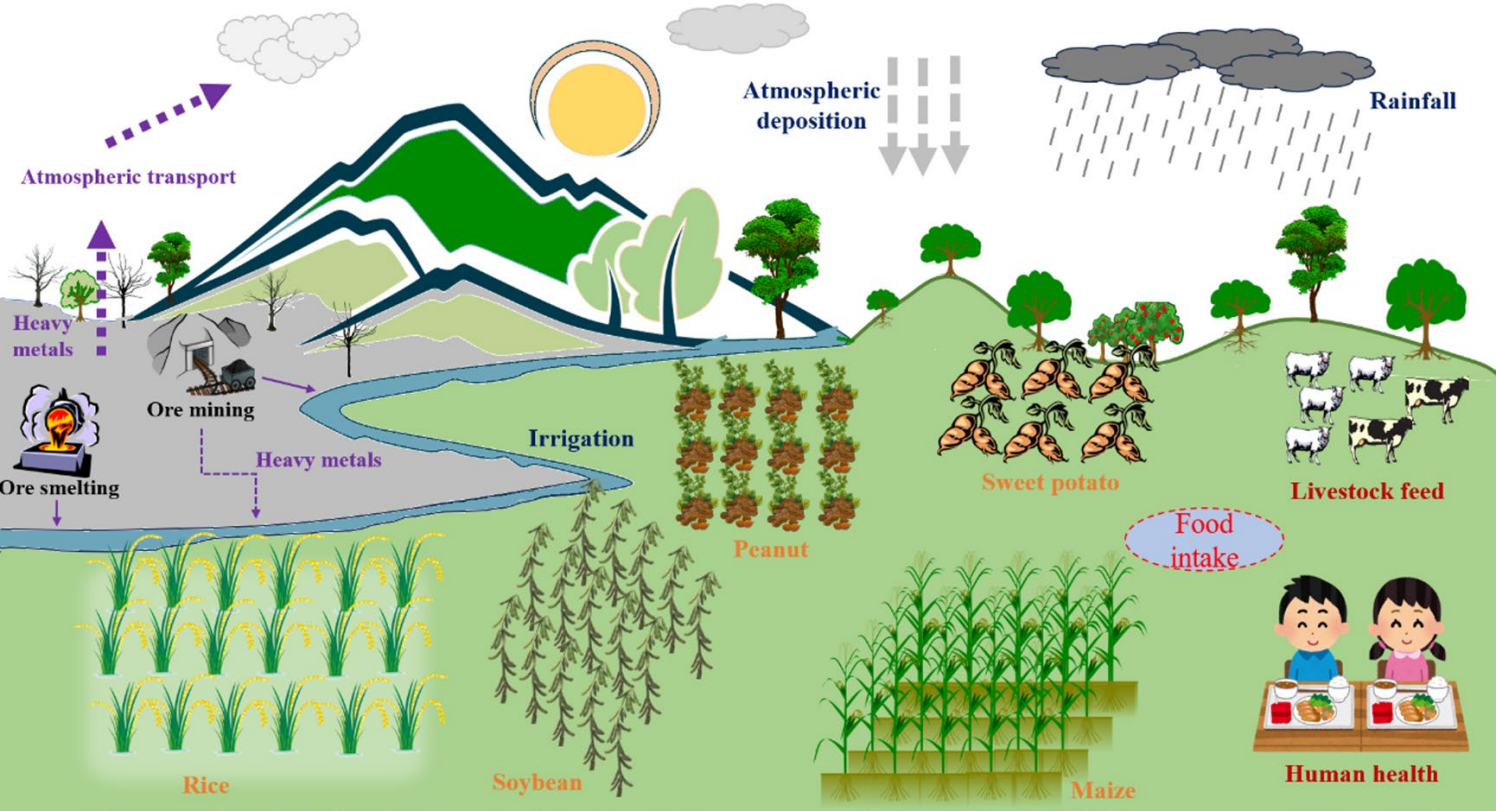
The impact of manganese mining and smelting on surrounding crop production and ecological health.

The main pathways for the spread of trace metals from manganese mining and smelting (a key economic activity in the study area) include pollution of the atmosphere, soil, and water, which are closely related to the ecological health and agricultural safety of the surrounding area (Fig. 9). Trace metals cause atmospheric pollution mainly by dust, metal particles, and aerosols, while polluted soils include mining processes, smelting processes, tailings piles, and rainwater washout^46^. Furthermore, surface water and groundwater are also highly susceptible to contamination, and these sources are often used for irrigation and drinking water^47^. Therefore, the assessment of ecological security and health risks of trace metals should include not only crop security but also drinking water security. The results of this study provide clear support for both hypotheses proposed in the Introduction. For Hypothesis 1, rice accumulated higher concentrations of Mn (2.27–3.37-fold higher than other crops), Cr (78.47 ± 27.11 mg/kg), and As (root concentration 49.80%), confirming its higher affinity for these metals. Additionally, trace metals (e.g., Mn, Cr, As, Pb) were predominantly sequestered in non-edible tissues (roots, leaves, shells) of all crops, with edible parts (fruits, roots of sweet potato) showing the lowest bioconcentration factors (0.01–0.05), which validates the “protective sequestration” mechanism for reproductive/edible organs. For Hypothesis 2, health risk assessment demonstrated that rice consumption posed potential chronic risks for adults (Cr-THQ = 3.09) and chronic toxic effects for children (As-THQ = 10.72), whereas sweet potato, peanut, soybean, and maize showed THQ < 1 for both populations. This confirms that rice is the primary contributor to trace metal-related health risks, especially for children with lower body weight and higher relative food intake. The present study found that rice is the most health-risky crop in the region, and the probable reason for this is that amounts of trace metals accumulate in the irrigation water, whereas the other crops are grown in dryland soils, which are effective in minimizing the harmful effects of trace metal exposure on the crops. Furthermore, this study found that although the fruit of the crops accumulated the lowest concentrations of trace metals, many of the stems and leaves of the crops are used as animal feed, and the resulting indirect effects on human health beings must also be emphasized. Therefore, to mitigate the impact of trace metals on crops in such mining areas, the following can be done. Strengthen environmental protection of mining and smelting processes at the source, including reducing solid particles and dust, avoiding groundwater pollution, and safely landfilling tailings. In crop cultivation, selecting clean sources of irrigation water reduces crop exposure to trace metals. Meanwhile, metal chelating agents or activated carbon can be added to heavily contaminated soils to reduce the accumulation of trace metals in crops^48^. Some studies have shown that the use of biochar can be effective in reducing trace metal pollution during rice cultivation^49^. In the future, we should not only focus on the safety of crops, but also on water safety and indirect impacts from livestock, and provide effective recommendations for food security and ecological health around mining areas.

### Limitations

Despite the comprehensive assessment of trace metal accumulation and associated ecological and health risks in five staple crops around the manganese mining area in Chongqing, this study has several inherent limitations that should be acknowledged. First, irrigation water, hypothesized as a key contamination vector for crops (especially rice), was not directly sampled due to logistical constraints, with its contribution inferred only through field surveys and literature references, which limits the precise quantification of its relative role in crop trace metal accumulation compared to soil and atmospheric deposition. Second, soil redox potential (Eh), a critical parameter regulating the mobility and bioavailability of trace metals such as As and Cr in anaerobic paddy systems and aerobic dryland soils, was not measured due to instrumentation limitations, hindering a mechanistic understanding of how soil redox conditions modulate trace metal translocation from soil to different crops. Third, the sampling design prioritized spatial coverage across 33 sites but resulted in unbalanced sample sizes among crops (e.g., *n* = 10 for peanut vs. *n* = 16 for rice), and post-hoc power analysis indicated that only strong effects (Cohen’s d > 1.2) could be reliably detected, potentially overlooking subtle differences in trace metal accumulation between certain crops; additionally, the cross-sectional sampling conducted once in 2020 lacks long-term dynamic data on seasonal variations in trace metal bioavailability and crop accumulation, which may limit the generalizability of the findings. Fourth, the health risk assessment adopted conservative simplifications, including assuming children’s crop intake as half of adults’ and using fixed body weight values, which may not fully reflect local dietary patterns—for instance, older children (7–12 years) in Chongqing often have higher rice consumption than the assumed 165 g/day—and the model did not account for multi-pathway exposure (e.g., drinking water, soil ingestion) or indirect risks from trace metal accumulation in livestock fed with crop stems and leaves, which may lead to underestimation of actual health risks in vulnerable populations. The updated health risk assessment adopts local dietary survey data and national growth standards to revise child exposure parameters (IR: 0.22–0.28 kg/d, BW: 22–28 kg), reducing the over-conservatism of previous assumptions. However, individual differences in children’s dietary habits (e.g., rice intake of 13–15-year-old adolescents) and multi-pathway exposure (drinking water, soil ingestion) were not fully considered, which may still lead to slight deviations in risk estimates – this will be addressed in future follow-up studies. These limitations do not undermine the core findings of the study, such as rice being the primary crop posing health risks and trace metals being preferentially sequestered in non-edible tissues of crops, but they highlight areas for refinement in future research to strengthen the scientific basis for soil remediation and food safety strategies in mining-impacted agricultural systems.

## Conclusions

This study investigated the accumulation of trace metals in crops and their ecological risks around a manganese mining area in Chongqing, China, with explicit connections to national and international food safety standards. Mn was the most abundant metallic element in all crops, with rice accumulating higher Mn concentrations than other crops, while Zn accumulation followed the order of peanut > soybean > maize > rice > sweet potato, and the cumulative concentrations of trace metals in crops were Cr > Cu > Ni > Pb > As > Sb. For the bioconcentration factor, Cd was the highest, mainly enriched in peanuts and soybeans, followed by Zn (concentrated in peanuts and soybeans) and Mn (preferred in rice and soybeans), with trace metals predominantly accumulating in roots and leaves and minimally in fruits, reflecting crops’ protective mechanisms for reproductive organs in line with the risk mitigation goals of food safety standards such as China’s GB 2762 − 2017 and international guidelines. The two hypotheses proposed in this study were fully verified: (1) Rice exhibited higher accumulation of Mn, Cr, and As compared to the other four crops, and trace metals were preferentially sequestered in non-edible tissues (roots, leaves) to protect edible parts, which is consistent with the self-protection mechanism of crops; (2) Rice posed greater human health risks, with Cr and As leading to chronic toxic effects in children, whereas other crops were safe for consumption. Ecological risk assessment showed that roots had the highest risk, followed by leaves and shells, with stems and fruits the lowest, and Cr, Ni, Pb, and As were the main pollutants causing severe crop pollution, with the overall pollution level being rice > maize > peanut > soybean > sweet potato. Health risk assessment confirmed that only rice posed potential health risks (Cr-THQ > 1 for adults) and chronic toxic effects (As-THQ > 10 for children), while sweet potato, peanut, soybean, and maize were safe for consumption, aligning with USEPA health risk benchmarks. These findings highlight that rice should be a priority monitoring target in line with national food safety supervision requirements, and promoting low-accumulating crops like sweet potato complies with international food safety risk management principles, providing scientific support for soil remediation and food security guarantee around mining areas that align with both national and international food safety standards.

## Data Availability

All data generated or analyzed during this study are included in this manuscript.

## Declarations

### Statement

The study complied with all relevant ethical guidelines for field sampling. We confirm that all plant and soil samples were collected from publicly accessible agricultural lands. No sampling occurred on private or restricted-access areas, and thus no landowner permissions were required for this study.
